# Genetics of Triglycerides and the Risk of Atherosclerosis

**DOI:** 10.1007/s11883-017-0667-9

**Published:** 2017-05-23

**Authors:** Jacqueline S. Dron, Robert A. Hegele

**Affiliations:** 1 Department of Biochemistry, Schulich School of Medicine and Dentistry, Western University, London, ON Canada; 2 Robarts Research Institute, Schulich School of Medicine and Dentistry, Western University, 4288A-1151 Richmond Street North, London, ON N6A 5B7 Canada; 3 Department of Medicine, Schulich School of Medicine and Dentistry, Western University, London, ON Canada

**Keywords:** Polygenic, Monogenic, Complex trait, DNA sequencing, Genetic association, Mendelian randomization

## Abstract

**Purpose of Review:**

Plasma triglycerides are routinely measured with a lipid profile, and elevated plasma triglycerides are commonly encountered in the clinic. The confounded nature of this trait, which is correlated with numerous other metabolic perturbations, including depressed high-density lipoprotein cholesterol (HDL-C), has thwarted efforts to directly implicate triglycerides as causal in atherogenesis. Human genetic approaches involving large-scale populations and high-throughput genomic assessment under a Mendelian randomization framework have undertaken to sort out questions of causality.

**Recent Findings:**

We review recent large-scale meta-analyses of cohorts and population-based sequencing studies designed to address whether common and rare variants in genes whose products are determinants of plasma triglycerides are also associated with clinical cardiovascular endpoints. The studied loci include genes encoding lipoprotein lipase and proteins that interact with it, such as apolipoprotein (apo) A-V, apo C-III and angiopoietin-like proteins 3 and 4, and common polymorphisms identified in genome-wide association studies.

**Summary:**

Triglyceride-raising variant alleles of these genes showed generally strong associations with clinical cardiovascular endpoints. However, in most cases, a second lipid disturbance—usually depressed HDL-C—was concurrently associated. While the findings collectively shift our understanding towards a potential causal role for triglycerides, we still cannot rule out the possibilities that triglycerides are a component of a joint phenotype with low HDL-C or that they are but markers of deeper causal metabolic disturbances that are not routinely measured in epidemiological-scale genetic studies.

## Introduction

An enduring controversy in the lipoprotein field has centered on the causal role of elevated plasma triglyceride (TG) levels in atherosclerotic cardiovascular disease (CVD) [1]. Are TG-rich lipoproteins the smoking gun or an innocent bystander in atherogenesis? TG undoubtedly keeps bad company: Elevated TG is a central feature of the atherogenic dyslipidemia complex [2], which encompasses depressed high-density lipoprotein (HDL) cholesterol (C) and elevated small dense low-density lipoprotein (LDL) particles, both of which are themselves plausibly causally related to CVD. High TG is further correlated with such potentially causative non-lipoprotein parameters as abdominal obesity, insulin resistance, hypertension, hepatosteatosis, low-grade inflammation, a pro-coagulant state, and perturbed endothelial function [3••]. It has been notoriously difficult to disentangle elevated TG levels from this complex network of confounding variables even in reductionist experimental systems based upon cellular mechanisms, epidemiological data, or clinical trials [4]. More recently, human genetic researchers have offered their tools to help clarify the controversy by attempting to isolate the effect of TG-rich lipoproteins on CVD risk [5]. But, while the picture is somewhat less hazy in the era of Mendelian randomization, we submit that definitive evidence for a direct causal role of TG in CVD still eludes our grasp. Not ruled out are the possibilities that TG forms a conjoint causal phenotype with low HDL-C or is merely the tip of an iceberg of unmeasured causal agents that are acting directly at the site of vascular pathology.

## Defining Hypertriglyceridemia

Hypertriglyceridemia is a commonly ascertained clinical phenotype. A proposed simplified definition of hypertriglyceridemia is based on somewhat arbitrary but clinically useful consensus thresholds [6]. A normal TG level is <2 mmol/L (<175 mg/dL); mild-to-moderate elevation is between 2 and 9.9 mmol/L (175 to 885 mg/dL), while a severe elevation is >10 mmol/L (>885 mg/dL) [6]. TG level measured statically, either fasting or especially in the non-fasting state, is an integrated marker for a large number of associated lipoprotein disturbances [1]. For example, TG is ferried within TG-rich lipoproteins, which in the fasting state are mainly very-low-density lipoproteins (VLDL) [6]. In the non-fasting state, chylomicrons are transiently elevated and contribute in varying degrees to elevated TG levels [1]. Furthermore, the TG contained within remnants of chylomicrons and VLDL, and also intermediate-density lipoproteins (IDL), contributes to the integrated total TG level. In addition, the cholesterol carried within non-HDL particles, which comprise all the above atherogenic lipoproteins plus Lp(a), and defined as total cholesterol minus HDL-C, is correlated with TG levels [7]. Apolipoprotein (apo) B levels are also elevated when TG is elevated [8]. In particular, in the post-prandial state, the intestinal form of apo B (B-48) is increased when chylomicrons and their remnants are increased [9], while the hepatic form of apo B (B-100) that characterizes VLDL particles fluctuates less in response to meals.

### Prevalence of Hypertriglyceridemia

The distribution of fasting TG levels in North America permits approximation of patient numbers with hypertriglyceridemia according to the clinical cut-points proposed above. From the Canadian Heart Health Surveys, the mean overall TG level in adults was 1.55 mmol/L [10]. A level of 2.0 mmol/L represents about the top 25th percentile, while a level of 3.3 mmol/L represents the top 5th percentile, and a level exceeding 10 mmol/L is seen in about one in 600 individuals [10, 11]. Assuming similar distributions of this trait in contemporary westernized societies, mild-to-moderate hypertriglyceridemia could be as prevalent as one in four people at the low-end of the definition. The traditional cut-point of the top 5th percentile that was used to define “hyperlipoproteinemia type 4” is 3.3 mmol/L. Severe hypertriglyceridemia >10 mmol/L has a population frequency somewhat lower than heterozygous familial hypercholesterolemia [12].

### Hypertriglyceridemia and Atherosclerosis

Using these definitions, the assimilated understanding of biochemistry is that for individuals with mild-to-moderate hypertriglyceridemia, the predominant lipoprotein disturbance is VLDL, their remnants, and IDL [13]. Chylomicrons can also be present in this TG range, but the preponderance of TG-rich lipoprotein species is of hepatic origin. These particles are atherogenic, although it is not their TG content that contributes to growth of the atherosclerotic plaque—the cholesterol carried within these TG-rich lipoproteins finds itself at the scene of the crime, within arterial wall foam cells and the evolving plaque [14]. This is a modernization of the seminal Zilversmit hypothesis, an early and prescient articulation of the atherogenic role of TG-rich lipoproteins [15]. According to this model, TG-rich lipoproteins are metabolically independent of LDL-C in atherogenesis and, in fact, act additively to further increase risk.

From the population distribution of TG levels, most patients with “hypertriglyceridemia” fall within the mild-to-moderate range, and thus any potential atherosclerosis risk is tied to primarily smaller TG-rich particles such as VLDL and IDL. At higher strata of TG levels, which become rarer as one ascends the Gaussian extreme rightwards, larger particles such as chylomicrons and their remnants begin to predominate. In this important but much less commonly encountered subgroup, it has been more or less axiomatic that chylomicrons are too large to penetrate the arterial wall [4, 16]. However, chylomicron remnants, especially on the smaller end of the spectrum, may contribute to atherogenesis [4].

Thus, among the diverse range of patients with severe hypertriglyceridemia, those with monogenic impairment of lipolysis (discussed below) would have primarily chylomicronemia, with minimal to no increase in remnants, mainly because the lipolytic deficiency prevents their generation. Atherosclerosis risk is relatively low in this situation. In contrast, among individuals with the same degree of TG elevation due to varied polygenic plus secondary factors, the spectrum of TG-rich particles is much more diffuse and includes many remnant particles, since lipolysis is not completely impaired [17]. Here, one could postulate that atherosclerosis risk is increased, due to the relative abundance of atherosclerosis-related remnants.

### Role of Genetics in Hypertriglyceridemia

Monogenic and polygenic factors contribute to both mild-to-moderate and severe hypertriglyceridemia; the relative burden of these factors together with secondary non-genetic factors can determine the severity of the phenotype [6, 18]. Elevated TG can result from either reduced catabolism or overproduction of TG-rich lipoproteins, each of which arises in turn depending on underlying genetic variation. The specific combination of qualitatively and quantitatively abnormal TG-rich lipoproteins distinguishes mild-to-moderate from severe hypertriglyceridemia [6, 18].

As mentioned, elevated VLDL is the predominant lipoprotein disturbance in individuals with TG levels between 2 and 9.9 mmol/L, while elevated chylomicrons start to contribute to TG >10 mmol/L [2]. Thus, factors related to biosynthesis, secretion, and catabolism of VLDL would be relatively more important in susceptibility to mild-to-moderate hypertriglyceridemia. In contrast, factors related to biosynthesis, secretion, and catabolism of chylomicrons are relatively more important in susceptibility to severe hypertriglyceridemia, although there is considerable overlap with factors that modulate VLDL levels, particularly on the catabolic side [18]. A corollary of this model is that individuals with polygenic severe hypertriglyceridemia have a greater burden of genetic susceptibility components than individuals with polygenic mild-to-moderate hypertriglyceridemia. Furthermore, secondary factors may be quantitatively and qualitatively more extreme in patients with severe compared to mild-to-moderate hypertriglyceridemia. Finally, control of secondary factors and medications might be relatively more efficacious in normalizing TG levels in individuals with polygenic mild-to-moderate hypertriglyceridemia than in those with severe hypertriglyceridemia. Both mild-to-moderate and severe hypertriglyceridemia are primarily polygenic traits, with the exception of the small subgroup with monogenic chylomicronemia due to deficiency of lipoprotein lipase (LPL) and related factors [6].

## Genetics of Severe Hypertriglyceridemia

As discussed above, severe hypertriglyceridemia is basically synonymous with “chylomicronemia” [13, 19]. Given their surface area-to-volume ratios, chylomicrons are essentially the only particle physiologically able to produce such extreme hypertriglyceridemia [13, 19]. If there is sufficient lipolytic capacity to generate chylomicron remnant particles, VLDL can be produced and VLDL remnants may also be elevated. But, this is not the case in monogenic disorders that shut down lipolysis. Thus, monogenic chylomicronemia is ultra-rare and is associated with a narrowly defined increase in chylomicrons and increased pancreatitis risk. In contrast, polygenic chylomicronemia is much more common and is associated with increased chylomicrons, VLDL, and remnants of both in addition to IDL. HDL-C is depressed in both circumstances [13, 19], although CVD risk is increased mainly in polygenic chylomicronemia.

### Rare Variants in Monogenic Chylomicronemia

Monogenic chylomicronemia is extremely rare in the population, appearing in one in 100,000–1,000,000 individuals [13, 19]. Clinical diagnosis can occur between infancy and early adulthood [13, 19]. With excess accumulation of chylomicrons, severe hypertriglyceridemia may be detected soon after birth. Chylomicronemia, but not elevated VLDL, produces plasma with a turbid, milk, or “lipemic” appearance. The main risk to health of elevated chylomicrons per se is not vascular disease, but rather acute pancreatitis, which can be life-threatening. Monogenic chylomicronemia patients also show, from a young age, failure to thrive and exhibit clinical manifestations including eruptive xanthomas, lipemia retinalis, hepatosplenomegaly, chronic abdominal pain, nausea, and vomiting [13, 19].

Monogenic chylomicronemia follows autosomal recessive inheritance of rare bi-allelic variants—either homozygous or compound heterozygous—disrupting one of five canonical genes involved in TG metabolism. These genes are as follows, in descending order of prevalence in monogenic chylomicronemia: *LPL* encoding LPL, *GPIHBP1* encoding glycosylphosphatidylinositol-anchored HDL-binding protein 1 (GPIHBP1), *APOC2* encoding apo C-II, *APOA5* encoding apo A-V, and *LMF1* encoding lipase maturation factor 1 (LMF1) [13]. Large-effect loss-of-function disruptions affecting these gene products cripple lipolysis, and in particular, catabolism of chylomicron particles. In up to 90% of monogenic chylomicronemia cases, *LPL* is the most commonly disrupted gene, with bi-allelic loss-of-function variants [20]. Under normal physiological conditions, LPL is anchored to the luminal surface of vascular networks traversing muscle and adipose tissue, where it comes into contact with circulating lipoproteins and helps to maintain TG levels [21]. LPL also interacts at various points in its life cycle with the products of the other minor genes that cause monogenic chylomicronemia.

In addition to the direct loss of function of the *LPL* gene, bi-allelic large-effect disruptions to the other four canonical genes also prevent the hydrolyzing action of LPL and result in the elevation of TG present in monogenic chylomicronemia. LMF1 acts as a chaperone to assist in the maturation of LPL, while GPIHBP1 is responsible for transporting and anchoring mature LPL to the vascular lumen surface, in which the lipase becomes fully activated [21]. Without the proper function of these two proteins, through the mechanisms previously described, reduced chylomicron catabolism results in severe hypertriglyceridemia. Apo C-II and apo A-V are present on chylomicrons and are necessary for the interaction between these TG-rich lipoproteins and LPL. Apo C-II is absolutely necessary for the particle’s interaction with LPL and the initiation of hydrolysis, while apo A-V interacts with GPIHBP1 to indirectly enhance the function of LPL, although its exact mechanism of action is still not fully understood. Similar to disruptions of *LMF1* and *GPHIBP1*, rare bi-allelic variants in *APOC2* and *APOA5* diminish the LPL-based catabolism of chylomicrons. Interestingly, autoantibodies can arise against either LPL [22] or GPIHBP1 [23•], on the background of normal gene structure, which also can lead to chylomicronemia. To date, ~100 families worldwide have been reported with monogenic chylomicronemia due to bi-allelic loss-of-function variants in these four crucial genes; any relationship with atherosclerosis is not definitive across these very rare families.

### Rare Variants in Polygenic Chylomicronemia

While monogenic chylomicronemia is extremely rare, it is much more likely to observe rare heterozygous variants in these genes in adult patients with severe hypertriglyceridemia. Rare heterozygous loss-of-function variants are an important genetic contributor to polygenic chylomicronemia. Most healthcare providers seem to recall the monogenic basis of severe hypertriglyceridemia but often retain the perception that every patient encountered with severe hypertriglyceridemia and pancreatitis must have one of these ultra-rare deficiencies. This perception was perpetuated because of the unavailability of testing for absent post-heparin lipase activity or mutations in causative genes, both of which could definitively rule out these monogenic disorders.

Although the degree of TG elevation is comparably severe in monogenic and polygenic chylomicronemia, there are key clinical and biochemical differences. Polygenic chylomicronemia is more often diagnosed in adulthood and is associated with a broader range of deleterious lipoprotein abnormalities (including elevated remnants and IDL), lower pancreatitis risk, and likely increased CVD risk [13, 19]. These clinical differences may be due to only partially disrupted lipolysis from heterozygosity for mutations in *LPL*, *GPIHBP1*, *APOC2*, *APOA5*, and *LMF1* [24, 25]. However, many heterozygotes for such dysfunctional mutations have a normal lipid profile [24, 25]; a secondary factor is required to force expression of the severe phenotype.

Not only are polygenic chylomicronemia patients more likely to carry disruptive heterozygous variants in these canonical genes, but they are also more likely to carry rare variants in non-canonical genes involved in TG metabolism [25–27]. For instance, *CREB3L3* encoding the transcription factor cyclic AMP-responsive element-binding protein H is an example of a gene that harbors rare large-effect determinants of human TG levels discovered through the use of animal models [28]. In addition, *GCKR* encoding glucokinase regulatory protein is an example of a gene that harbors rare large-effect determinants of human TG levels that was initially identified as a common locus for TG levels through large-scale genome-wide association studies (GWAS) [29]. Therefore, the list of rare heterozygous large-effect variants underlying severe hypertriglyceridemia is extensive.

### Common Small-Effect Variants in Polygenic Chylomicronemia

In addition to the accumulation of rare heterozygous variants within TG-related genes, another defining genetic feature of polygenic chylomicronemia is the increased burden of common single nucleotide polymorphisms (SNPs) associated with TG levels [30, 31]. These variants are common in the general population and have small phenotypic effects, with mild influences on TG levels. GWAS have identified 185 SNP loci associated with circulating levels of lipids and lipoproteins; of these SNPs, >30 have a primary association with TG [32]. Essentially all reported SNPs also have associations with multiple lipoprotein traits, typically a reciprocal relationship between TG and HDL-C. This biological reality makes it challenging to isolate genetic influences on TG only and to draw conclusions when these markers are used as instruments in the Mendelian randomization studies discussed below; it is difficult to dissociate the effect of elevated TG from concurrently depressed HDL-C in gauging causality in atherosclerosis.

Several GWAS-identified SNPs are within loci already known from classical biochemistry and cell biology to be involved in TG metabolism, including *LPL* and *APOA5* [33]. Others were found in close proximity to genes that at the time were not relevant but were found to be in subsequent studies (i.e. *GCKR*), and many SNPs identified are intergenic and may be important in regulatory processes [32]. When considering TG-associated SNPs in polygenic chylomicronemia patients compared to normolipidemic individuals, a distinct increase in SNP accumulation in these patients has been observed and quantified using polygenic risk scores [25, 26]. Individually, each SNP has a slight influence on TG levels; however, when a substantial burden of multiple small-effect variants is present in an individual, it can synergistically contribute towards an overall large phenotypic effect. There are some recent examples of patients with chylomicronemia who also had a high polygenic risk score for TG [34, 35].

The contributory effects coming from rare heterozygous variants with larger phenotypic influences, and the excessive accumulation of common variants scattered throughout the genome, all work in concert to produce polygenic chylomicronemia, including severe hypertriglyceridemia due to perturbations of chylomicrons, as well as other TG-rich lipoproteins.

## Genetics of Mild-to-Moderate Hypertriglyceridemia

The same general architecture of genetic susceptibility is seen in patients with mild-to-moderate hypertriglyceridemia as in patients with severe hypertriglyceridemia [25]. Specifically, the pool of patients with the milder hypertriglyceridemia phenotype formerly known as Fredrickson type 4 (here considered equivalent to “mild-to-moderate hypertriglyceridemia”) also shows enrichment of rare heterozygous large-effect variants and common small-effect SNP loci bundled into a polygenic risk score, although not as extreme as Fredrickson type 5 (here considered equivalent to “severe hypertriglyceridemia”) [25]. These findings need to be replicated but suggest that hypertriglyceridemia along its spectrum of severity is a polygenic trait with similar genetic susceptibility components, both common and rare. Furthermore, final clinical expression of the phenotype—i.e. mild-to-moderate or severe—is related to qualitative and quantitative differences in the precise mixture of susceptibility variants in an individual’s genome. A higher burden of both rare and common TG-raising variants would be associated with a more extreme phenotype, such as polygenic chylomicronemia. Importantly, secondary non-genetic factors, including diet, alcohol intake, obesity, diabetes control, liver, and renal disease, are at least as important as genetic susceptibility in determining the final quantitative TG phenotype [6].

## Genetics of Low Triglyceride Levels: Familial Hypotriglyceridemia

For completeness, we briefly mention familial hypotriglyceridemia, defined as very low or absent TG levels due to various genetic factors. The interested reader is referred to a more thorough review of this topic [11]. As with familial hypertriglyceridemia, genetic determinants of hypotriglyceridemia include ultra-rare monogenic syndromic disorders that are associated with a range of other lipoprotein, biochemical and clinical abnormalities, such as abetalipoproteinemia and homozygous hypobetalipoproteinemia, which result, respectively, from bi-allelic mutations in *MTTP* and *APOB* genes encoding, respectively, microsomal TG transfer protein, and apo B [36]. Other informative conditions, mainly characterized by multiple biochemical disturbances including very low TG levels, include deficiencies of apo C-III and angiopoietin-like protein 3, which result from bi-allelic mutations in *APOC3* and *ANGPTL3* genes, respectively [37, 38]. Heterozygotes for *MTTP* loss-of-function variants have no obvious clinical or biochemical phenotypes, while heterozygotes for the other three deficiencies have depressed levels of TG and other lipoprotein traits, including LDL-C. Observational studies in these families suggest reduced risk of atherosclerosis, although these findings are somewhat limited by incomplete ascertainment and relatively small sample sizes.

## Genetic Evidence for Association Between Triglycerides and Atherosclerosis

The genetic evidence linking TG levels and atherosclerosis risk has been reviewed recently, from the perspective of LPL biology [39•]. Both common and rare genetic variants that are associated with plasma TG levels in populations can be tested for their association with atherosclerosis endpoints, such as coronary heart disease (CHD) using meta-analyses of several cohorts within the Mendelian randomization framework [40]. Under this construct, if a genetic variant associated with TG is also associated with the clinical endpoint, the conventional interpretation is that TG itself is directly associated with the outcome. However, in the case of TG levels, almost all significantly associated genetic variants—both common and rare, both large-effect and small-effect—are concurrently associated with at least one other lipid trait, usually reduced HDL-C (see Table 1). In the recent reporting of these studies, investigators tend to emphasize the directionally consistent genetic association between elevated TG and elevated clinical endpoints. However, virtually, none of these variants individually or in combination is associated with TG in a completely isolated fashion.

**Table 1:** Selected genetic factors and their association between lipid and lipoprotein levels, and coronary artery disease

Gene and variant(s)	Number of patients	Measures	Outcome metric	*P* value	Independent TG effect on CAD?	Reference
GWAS results						
185 lipid-associated SNPs	86,995	CAD (TG predictor)	0.36β (0.057 SEM)	1 × 10^−9^	Probably	[41]
		CAD (HDL-C predictor)	−0.04β (0.037 SEM)	0.35		
		CAD (LDL-C predictor)	0.38β (0.034 SEM)	2 × 10^−22^		
*ANGPTL4*						
E40K (rs116843064)	42,855	TG	13% ↓ levels	2.0 × 10^−23^	No	[42•]
		HDL-C	7% ↑ levels	1.6 × 10^−17^		
		LDL-C	↑ 1.3 mg/dL	NS		
		TC	↓ 0.2 mg/dL	NS		
		CAD	OR 0.81	0.002		
	193,638	CAD	OR 0.86	4.0 × 10^−8^	No	[43•]
	10,088	TG	↓ 0.335	1.6 × 10^−13^		
		HDL-C	↑ 0.295	8.2 × 10^−11^		
		LDL-C	↓ 0.064	0.16		
	80,111	TG	11.9% ↓ levels	1.4 × 10^−30^	No	[44•]
	119,514	HDL-C	↑ 0.10 mmol/L	3.9 × 10^−23^		
	119,146	non-HDL-C	↓ 0.16 mmol/L	8.7 × 10^−11^		
	269,344	CAD	OR 0.80	3.4 × 10^−6^		
Rare inactivating variants	41,252	TG	13% ↓ levels	0.02	No	[42•]
		HDL-C	9% ↑ levels	0.009		
		LDL-C	↑ 2.4 mg/dL	NS		
		TC	↓ 1.0 mg/dL	NS		
		CAD	OR 0.56	0.05		
Any of 10 rare variants	13,758	TG	35% ↓ levels	0.003	Possible	[43•]
		HDL-C	↑ 4.77 mg/dL	0.19		
		LDL-C	↓ 11.53 mg/dL	0.3		
		CAD	OR 0.47	0.04		
*APOA5*						
Rare nonsynonymous variants	13,432	Early-onset MI or CAD	OR 2.2	5 × 10^−7^	No	[45]
	2830	TG	60.7% ↑ levels*	0.007		
		HDL-C	25.2% ↓ levels*	0.007		
		LDL-C	1.8% ↑ levels*	0.66		
Rare deleterious (PolyPhen)	13,432	Early-onset MI or CAD	OR 2.0	6 × 10^−5^		
Rare deleterious (broad)			OR 2.2	2 × 10^−5^		
Rare deleterious (strict)			OR 3.3	0.008		
Rare disruptive			OR 4.5	0.007		
*APOC3*						
Any of three rare variants	75,725	TG	44% ↓ levels	<0.001	No	[46]
		Ischemic vascular disease	HR 0.59	0.007		
		Ischemic heart disease	HR 0.64	0.04		
Any of four rare variants	41,671	TG	39% ↓ levels	<1 × 10^−20^	No	[47]
		HDL-C	↑ 10.8 mg/dL	<1 × 10^−20^		
		LDL-C	↓ 3.8 mg/dL	0.19		
	110,970	CHD	OR 0.60	4 × 10^−6^		
Any of seven rare variants	3734	TG	38.5% ↓ levels	6 × 10^−9^		
		HDL-C	22.1% ↑ levels	4 × 10^−6^		
		LDL-C	16.0% ↓ levels	0.05		
*ASGR1*						
del12 in intron 4	99,893	TG	6.3% ↓ levels	0.003	No	[48•]
	140,889	HDL-C	↑ 2.5 mg/dL	3.9 × 10^−4^		
	140,521	non-HDL-C	↓ 15.3 mg/dL	1 × 10^−16^		
	73,542	LDL-C	↓ 12.5 mg/dL	3.9 × 10^−11^		
	291,938	CAD	OR 0.66	4.0 × 10^−6^		
W158X	8453	non-HDL-C	↓ 24.9 mg/dL	1.8 × 10^−3^	No	
		CAD	OR 0.65	0.24		
*LPL*						
D36N (also known as D9N; rs1801177)	10,208	TG	9% ↓ levels	0.005		[49]
	193,638	CAD	OR 1.13	2.0 × 10^−4^	NA	[43•]
	426,299	TG	↑ 10.3 mg/dL	<5 × 10^−8^		[50]
G215E (also known as G188E; rs118204057)	10,208	TG	26% ↓ levels	0.04		[49]
N318S (also known as N291S; rs268)	10,208	TG	13% ↓ levels	0.001		[49]
	426,299	TG	↑ 14.7 mg/dL	<5 × 10^−8^		[50]
S474X (also known as S447X; rs328)	10,208	TG	22% ↓ levels	<0.001		[49]
	193,638	CAD	OR 0.94	2.5 × 10^−7^	NA	[43•]
	426,299	TG	↓ 11.2 mg/dL	<5 × 10^−8^		[50]
Zero to three alleles (S447X, N291S, D9N, G188E)	10,208	TG	No change	<0.001	No	[49]
		HDL-C	No change	<0.001		
		Mortality from CVD	HR 0	NA*		
Four alleles (S447X, N291S, D9N, G188E)	10,208	TG	12% ↓ levels	<0.001	No	
		HDL-C	8% ↑ levels	<0.001		
		Mortality from CVD	HR 0.86	NA*		
Five alleles (S447X, N291S, D9N, G188E)	10,208	TG	21% ↓ levels	<0.001	No	
		HDL-C	12% ↑ levels	<0.001		
		Mortality from CVD	HR 0.81	NA*		
Six alleles (S447X, N291S, D9N, G188E)	10,208	TG	31% ↓ levels	<0.001	No	
		HDL-C	15% ↑ levels	<0.001		
		Mortality from CVD	HR 0.77	NA*		
rs301	426,299	TG	↓ 7.3 mg/dL	<5 × 10^−8^		[50]
rs326	426,299	TG	↓ 6.8 mg/dL	<5 × 10^−8^		
rs10096633	426,299	TG	↓ 9.3 mg/dL	<5 × 10^−8^		
All six combined (rs1081177, rs268, rs301, rs326, rs328, rs10096633)	426,299	CAD	OR 1.51	1.1 × 10^−22^	NA	
Any of 52 rare damaging variants	33,378	TG	↑ 19.6 mg/dL	0.01	No	
	32,580	HDL-C	↓ 3.6 mg/dL	0.001		
	31,549	Remnant cholesterol	↑ 5.6 mg/dL	0.001		
	46,891	CAD	OR 1.84	<0.001		
*SVEP1*						
D2702G (rs111245230)	193,638	CAD	OR 1.14	4.2 × 10^−10^	No	[43•]
	10,088	TG	↑ 0.05 SD units	0.19		
		HDL-C	↑ 0.023 SD units	0.56		
		LDL-C	↑ 0.011 SD units	0.78		

*CAD* coronary artery disease, *CVD* cardiovascular disease, *GWAS* genome-wide association study, *HDL-C* high-density lipoprotein cholesterol, *HR* hazard ratio, *LDL-C* low-density lipoprotein cholesterol, *MI* myocardial infarction, *NA* not applicable (for CAD endpoint, this indicates that the full lipid profile was not available), *NS* not significant, *OR* odds ratio, *SD* standard deviation, *SEM* standard error, *SNP* single nucleotide polymorphism, *TC* total cholesterol, *TG* triglycerides

*P values for these metrics were not available in text by Thomsen et al. [49]

**Percent change calculations based on the median lipid levels for non-carrier and carrier groups, provided by Do et al. [45].

So, even with this mountain of evidence purporting to show a causal relationship of TG with atherosclerosis, the possible involvement of correlated trait, usually low HDL-C, cannot be ruled out. While not explicitly stated, emphasis on TG may be due to a systematic unconscious bias that has arisen against HDL over the past decade for various reasons [51, 52]. HDL has fallen upon hard times in terms of consideration of its direct role in atherosclerosis risk; recent studies of variants that jointly influence TG and HDL-C tend to focus on the TG component.

### Common Variants Associated with Triglycerides and Atherosclerosis Risk

For instance, common *APOA5* variants were associated with both higher TG and increased CHD risk but were concurrently associated with lower HDL-C levels [53]. Furthermore, the common *LPL* p.S474X (also known as p.S447X) gain-of-function variant has long been associated with reduced TG, increased HDL-C, and reduced CHD risk in small cohorts [54], while the relatively common *LPL* p.D36N loss-of-function variant has been associated not only with increased TG and increased CHD risk but also with reduced HDL-C [53]. Associations of these two *LPL* variants with the joint high TG/low HDL-C atherogenic dyslipidemia complex and with CHD risk were recently confirmed in a large case-control sample [43•]. Another recent study that combined epidemiological samples and electronic health records analyzed common *LPL* variants and reported an odds ratio for CHD of 1.51 per one standard deviation of genetically determined increased TG, although these well-known variants—including LPL p.S474X, p.D36N and p.N318S—also have reciprocal effects on HDL-C levels, which were not reported in that paper [50]. Another study used multivariate regression to isolate the genetic effect on HDL-C and CHD risk; rather complicated statistical models showed that combinations of genetic determinants with predominantly TG-related effects were correlated with increased CHD risk, while combinations of genetic determinants with predominantly HDL-C-related effects were not [41]. However, almost all other studies of common variants and CHD risk have not been able to dissociate TG from HDL-C effects.

### Rare Variants Associated with Triglycerides and Atherosclerosis Risk

Similar inability to unscramble joint inverse effects on TG and HDL-C is seen in studies of rare genetic variants. For instance, massive high-throughput sequencing efforts showed that rare heterozygous loss-of-function *APOC3* mutations are primarily associated with reduced plasma TG levels: Mutation carriers had significantly reduced CHD risk, again supporting the idea that TG might contribute directly to atherosclerosis [46, 47]. However, these rare variants were almost always associated with reduced LDL-C and increased HDL-C [46, 47]. In addition, carriers of rare heterozygous loss-of-function mutations in *APOA5* that increased plasma TG levels had a twofold increased risk of early CHD [45], but these variants were also associated with increased LDL-C and decreased HDL-C. Furthermore, three recent studies of common and rare *ANGPTL4* loss-of-function variants associated with lower TG and higher HDL-C showed associations with reduced cardiovascular risk [42•, 43•, 44•]. Again, risk modulation was attributed to TG effects, despite the significant impact on HDL-C. The apparent protective effect of high HDL-C in the context of low TG cannot seem to be disentangled in these genetic experiments [42•, 43•, 44•].

There are more examples. Among 46,891 individuals with *LPL* gene sequencing data available, one in ~250 had a damaging rare mutation in *LPL* [50]. The authors found ~15% higher corrected TG levels in these individuals, although HDL-C was concurrently reduced by ~10%, and calculated remnant cholesterol was also increased by ~10%. Compared with non-carriers, heterozygous carriers had more CHD (odds ratio = 1.84 *P* < 0.001) [50]. Again, the authors focused almost entirely on levels of TG and remnant cholesterol, even though the latter was mathematically derived from TG and was thus highly correlated with it. In another study using a Mendelian randomization design, heterozygous carriers of rare *ANGPTL3* loss-of-function mutations, seen in one in ~300 people, had 17% and 12% reductions in plasma TG and LDL-C levels, compared with non-carrier controls; in this instance, HDL-C was not increased [55]. Carrier status was associated with a 34% reduction in odds of CHD (*P* = 0.04), although admittedly, this was a rather borderline association for such a gargantuan sample size. Again, although TG showed the greatest perturbation as a result of the genetic variation, a possible additive or potentiating effect of concurrent reduced LDL-C could not be excluded [55]. Another example of the confounding and interrelationship between lipoprotein and lipid traits was seen in a study of Icelanders, in which a rare loss-of-function variant of *ASGR1*, encoding an asialoglycoprotein receptor, had reduced TG and non-HDL-C, and increased HDL-C, together with 34% reduced CAD risk [48•]. Of course, non-HDL-C is a derived variable that does not represent an exclusive biological entity; by analogy, perhaps total plasma TG is a non-specific integrated trait that is either the composite of or an indirect marker for other bioactive components, some of which may directly act on the vascular wall in atherogenesis.

## Conclusion

Thus, plasma TG is a confounded metabolic variable or biomarker. The integrated overview is that atherosclerosis risk is elevated in individuals with mild-to-moderate hypertriglyceridemia, a largely polygenic trait. In mild-to-moderate hypertriglyceridemia, cholesterol for arterial plaque formation is contributed from VLDL, their remnants, and IDL. Among rarer individuals with severe hypertriglyceridemia, atherosclerosis risk would be increased among individuals with a diffuse spectrum of large TG-rich chylomicron remnants (i.e. in polygenic chylomicronemia) but probably not among those with chylomicrons predominantly (i.e. in monogenic chylomicronemia). The association of elevated TG levels with atherosclerosis is consistent from epidemiologic, mechanistic, and clinical trials, and the more recent genetic findings seem to be compelling. However, it continues to be challenging to disentangle the effects of TG from HDL-C. TG-raising variant alleles of several genes have shown generally strong associations with clinical CVD endpoints; however, in almost every case, a second lipid disturbance—usually depressed HDL-C—was concurrently associated, as outlined in Table 1. While the findings collectively shift our understanding to implicate TG towards causality, we still cannot rule out the possibilities that TG are a component of a joint phenotype with low HDL-C or that they are markers of deeper metabolic disturbances that are not routinely measured in epidemiological-scale genetic studies. Finally, the presence of unmeasured secondary factors, both lipid-related and non-lipid-related, would further contribute to—or perhaps primarily explain—increased atherosclerosis risk in genetically predisposed individuals with higher TG levels.
